# Does Experience Make Hucul Horses More Resistant to Stress? A Pilot Study

**DOI:** 10.3390/ani11123345

**Published:** 2021-11-24

**Authors:** Jadwiga Topczewska, Wanda Krupa, Zofia Sokołowicz, Jadwiga Lechowska

**Affiliations:** 1 College of Natural Sciences, University of Rzeszów, Zelwerowicza Street 4, 35-601 Rzeszow, Poland; zosokolo@ur.edu.pl (Z.S.); jlechowska@ur.edu.pl (J.L.); 2 Department of Animal Ethology and Wildlife Management, University of Life Sciences in Lublin, Akademicka Street 13, 20-950 Lublin, Poland; wanda.krupa@up.lublin.pl

**Keywords:** horses, saliva cortisol, stress response, performance assessment

## Abstract

**Simple Summary:**

Breeding programs dedicated to some horse breeds require the application of performance tests, which can be a source of stress exerting a negative impact on the welfare of these animals. By monitoring the level of stress with non-invasive methods, factors that reduce welfare and are not indispensable in horse breeding and use can be eliminated. An attempt was made to investigate whether the age and the number of starts in championships contribute to horses’ experience and therefore increase their resistance to stress. The results of the tests conducted on Hucul mares who participated in the Polish Championships for Hucul Horse showed an increase in cortisol levels in all mares after each element of the championship. Age and a higher number of horse starts did not result in increased resistance to stress accompanying the competition.

**Abstract:**

The aim of the study was to determine whether experience related to horse age and number of starts in championships influences stress level, measured by salivary cortisol concentration. The study involved 18 clinically healthy Hucul mares who participated in the Polish Championships for Hucul Horses. Evaluation of performance value was carried out in accordance with the guidelines specified in the breeding and genetic resources conservation program for this breed. The championship lasted two days, consisting of conformation evaluation, endurance, and Hucul path. Saliva was collected at baseline (T1), after arena assessment (T2), after endurance (T3), and on the second day after the Hucul path (T4). Cortisol levels increased from an average of 2.73 ± 1.18 ng/mL (T1) to 10.46 ± 8.03 ng/mL after T3. Significantly lower levels of free cortisol were detected in the saliva of the younger mares, up to 9 years old, and mares who participated in only one qualifying path after each element of the championship. The highest levels of cortisol (T3) were found in mares competing repeatedly on the qualifying path. No correlation was found between cortisol levels and the championship results. Participation of mares in the championship was associated with stress, which was reflected in the increase in cortisol levels in saliva.

## 1. Introduction

To assess the occurrence of stress in horses, the concentration of pituitary–adrenal axis hormones determined in saliva is increasingly used [1,2,3,4,5,6,7,8]. Non-invasive methods for assessment of stress response increasing, e.g., the level of free transcortin-unbound cortisol [9,10,11], are used in other animal species as well [12,13,14,15,16]. Such biological material is sampled with no additional pain, stress, or discomfort and the material can be collected by non-veterinarians [9,11,17]. In turn, the analysis of physiological markers and blood sampling require the work of trained personnel and is invasive. Therefore, determination of the level of cortisol in saliva seems to be an optimal solution, as it facilitates non-invasive assessment of the impact of stress factors on the animal. Theoretically, it may be assumed that, through stimulation of the autonomic nervous system, stress will have a mobilizing effect on a horse’s performance, which is important in terms of expected results. In a study by Peeters et al. [3], they found that horses that had higher salivary cortisol levels performed better. Similarly, Munk et al. [5] found that salivary cortisol levels can be positively correlated with performance. Cortisol concentrations were higher during competition and performance in one competition was positively correlated with baseline cortisol levels [5]. However, as demonstrated by Witkowska-Piłaszewicz et al. [18], the best racehorses exhibited a small increase in cortisol levels. Additionally, long-term stimulation of the hypothalamic–pituitary–adrenal axis (HPA) combined with the release of stress hormones may consequently lead to suppression of the immune system. Changes induced by the action of various environmental stimuli also influence the activity of the adrenal cortex and disrupt homeostasis in the organism [19,20]. At the same time, Pawluski et al. [19] found low cortisol levels in the evening when stress was chronic. Additionally, Fureix et al. [21] indicated low cortisol levels in the case of chronic stress.

Contemporary forms of the use of horses are often associated with intense physical exertion and exposure to unknown stimuli [9,22,23]. This applies not only to sports horses but also to those used in recreation [7], including breeds whose performance value must be assessed in accordance with the applicable breeding regulations. Their owners often attempt to achieve the best possible results by frequent participation in organized championships [24].

Hucul horses represent primitive breeds, which are currently used mainly in recreation, mountain tourism, and hippotherapy [25,26]. Hucul horses are one of the oldest mountain horse breeds bred in the Hucul region of the eastern Carpathians. According to many researchers [27,28,29], they arose as a result of the cross-breeding of different types and breeds of horses; however, the most important factor in the development of their traits were the living conditions, mainly harsh climate, difficult mountainous terrain, and natural rearing on high mountain pastures. They were used mainly as saddle horses and pack horses. According to the Hucul Breeding Program [30], the height at the withers of adult stallions should be between 135 and 145 cm; of the mares between 132 and 143 cm measured with a stick, the chest circumference should be greater by at least 30 cm than the height at withers. The front circumference should be 17 to 20 cm in stallions and 16 to 19 cm in mares, measured at 1/3 of the height of the upper metacarpal bone at its thinnest point. Hucul horses should have a bay, gray, mousey, or buff coat, combined with a piebald tobiano pattern. They are characterized by a stocky build, considerably slower growth rate, and later somatic maturity than other breeds. The average body weight of Hucul horses found in the study by Łuszczyński et al. [31] was 463 kg. They are protected by the genetic resources conservation program [30], which in the case of the population in Poland, is associated with the possibility of obtaining financial support. This program also requires the assessment of the breeding and performance value of horses of this breed, being an indispensable element for breeding. Its formula usually facilitates evaluation of the breed-related predisposition. A passing grade in all elements indicates compliance with the type of breed, the correct conformation and correctness of movement, the proper preparation of the horse for riding work, strength and training, and ease in overcoming obstacles in the field. According to the breeding program, the Polish Championships for Hucul Horses is a facultative test of its performance value. Besides, from a conformation assessment, it includes a Hucul path with natural and artificial obstacles, typically present on routes for horse endurance and mountain rides. These are, for example, a steep descent, a footbridge over a ditch, a passage through water, or a tight passage. Although Hucul horses are believed to be intelligent and they willingly cooperate with humans [28,29], their participation in competitions may be associated with the risk of a temporary or long-term deterioration of welfare [18]. Experience is thought to exert a modifying effect on the occurrence of stress [18]; however, there are no studies confirming such claims in breeds that are not used in equestrian sports but are subjected to performance value assessment and when riders are not professionals.

The aim of the study was to determine the impact of the experience of Hucul horses participating in the Polish Championships for Hucul Horse, resulting from age and number of previous starts: (1) concentration of free cortisol in saliva as an indicator of stress, and (2) the relationship between cortisol concentration and the results obtained in the championships of horses of this breed.

## 2. Material and Methods

### 2.1. Animals

All examinations performed in this study are non-invasive and are routinely performed as part of everyday veterinary and management procedures. Following the existing law applicable in Poland, based on the Experiments on Animals Act from 15 January 2015 (Journal of Laws of the Republic of Poland, 2015, item. 266), non-invasive clinical studies do not require ethical approval.

The research material was collected during the Polish Championships for Hucul Horse held in September 2019 at the State Hucul Horse Stud in Regietów, where the largest European population of this breed is kept. The study involved 18 clinically healthy Hucul mares participating in the Polish Championships for Hucul Horse, aged from 5 to 14 years (9.05 ± 2.65), for which a complete set of results was collected, including positive completion of all elements of the competitions. In the Polish Championships for Hucul Horses, 40 horses took part, that is, 13 stallions, 25 mares, and 2 geldings. From a total of 40 horses (13 stallions, 25 mares, 2 geldings) that participated in the Hucul Horse Championship, 10 did not finish the competition. The reasons for elimination included a high heart above 64 beats/min a horse at the vet check point. The assessed lameness was as gait regularity and/or visible lameness, which was evaluated after the endurance–condition test and during the whole competition. According to the results of the competition, in the case of the Hucul path, a “route mistake” results in elimination. This was the case for 3 pairs of horse/rider. In case of one pair, it was a “resignation”/“withdraw the horse from competition”, and thus it was written in the results of the Hucul path. The assessed lameness was as gait regularity and/or visible lameness, which was evaluated after the endurance test and during the whole competition. All phases of the championship were completed by 27 horses, including 19 mares, 7 stallions, and one gelding. Saliva was collected from all horses if only the owners agreed (4 declined). As a result of the study, complete results were obtained, including both the completion of all elements of the championship and the amount of saliva that allowed the determination of cortisol concentration with laboratory procedures of 18 mares (95%). Animals were divided into two groups according to the mare’s age (9 horses ≤9 years, 9 horses ≥10 years), and into two other groups according to the number of starts on the qualifying path (6 horses starting only once, 12 horses starting ≥2 times).

The Polish Championships for Hucul Horses has been carried out for 25 years as a performance value assessment competition. It consists of (1) breeding assessment of the exterior and movement at walk and trot (B), (2) endurance–condition test (C), and (3) Hucul path (E).

For horses to participate in the Polish Championships for Hucul Horse, they must first qualify, which in turn depends on the horse’s ranking based on the results obtained in 7 qualification paths. In 2019, the horses were qualified between 25–26 May and 24–25 August. The qualification path consists of breeding assessment and Hucul path. According to the regulations [24], the number of starts of the horse in qualifications is not limited, however but there are restrictions on the number of starts per day in the Polish Championships for Hucul Horses. This restriction is due to additional sporting competitions taking place during the championships.

**Figure 1:**
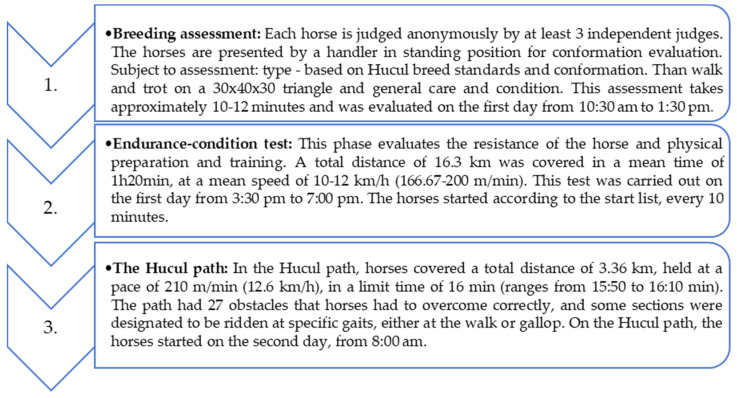
A diagram showing the order of competition during the Polish Championships for Hucul Horse in 2019.

During the breeding assessment (B) of exterior and movement, the horses were presented by the handler on the arena in the standing position and at walk and trot in the show arena, i.e., a 30 × 40 × 30 m triangle (Figure 1). Breeding assessment takes approximately 10–12 min. The race (C), i.e., the endurance–condition test, assesses horse’s endurance and preparation. It was carried out over a distance of 16.3 km within the standard time at a speed of 10–12 km/h (166.67–200 m/min). A total distance of 16.3 km was covered in a mean time of 1 h 20 min, according to the regulations of the 2019 championships. After crossing the finish line, and within a maximum time of 20 min, horses went a veterinary inspection that includes HR (64 beats/min maximum), dehydration rate, normal walking, vascular filling, and intestinal peristalsis. Any irregularities or abnormal parameters presented in horses resulted in the elimination of the animal. The Hucul path (E) covered a distance of 3360 m and was held at a pace of 210 m/min (12.6 km/h), with a time limit of 16 min (the norm ranges from 15:50 to 16:10). In addition to 27 obstacles to be overcome correctly, there were sections along the path to be covered at walk (twice) and canter (Figure 1). The rules for covering the route were defined in detail by the regulations.

Before the start of the competition on the first day, an obligatory veterinary overview of the horses takes place, during which physiological parameters, including heart rate (A), are determined. Baseline saliva (T1) samples are taken approximately 20 min after the examination. In the breeding assessment (B), the maximum score is 50 points. For each trait (type, conformation, walk, trot, and general care and condition) a horse can obtain a maximum of 10 points, with an accuracy of 0.5 for each trait. For the completed endurance–condition test (C) and the Hucul path (E), the horse can obtain a maximum of 80 points each. According to the regulations of the Polish Championships for Hucul Horses, the maximum number of bonus points possible to gain for all competitions is 210 (G). For points for the exterior score, a conversion factor of 0.8 is used, for the Hucul path score—1.25 and for the endurance test—0.75. At the same time, after each competition, the detailed results of each horse are given. During the research, average speeds (D and F) were calculated. They are made available (Data Availability Statement).

The weather on both competition days was similar, with a temperature of 14–16 °C, air humidity 75%, atmospheric pressure 1012 hPa, sun, wind speed 6 km/h.

### 2.2. Saliva Sampling

All saliva samples were collected using Salivettes cellulose swabs (Sarstedt, Nümbrecht, Germany) and placed under and over the tongue for approximately 60 s. The sampling procedure did not require restraining the horses and did not produce any discomfort in the animals. Saliva samples were collected four times: T1. Baseline: samples were taken from 9:00 to 10:00 a.m., 30 min before the competition starts. T2. Post breeding assessment: sample was taken 10 min after judges completed the evaluation. T3. After endurance–condition test: saliva was obtained from 5:00 to 6:50 p.m., after the vet examination 10 min approximately after. T4. After the Hucul path: Samples were obtained once the horses finished the Hucul path, approximately 10 min after. The Hucul path (T4) competition took place from 8:00 to 12:00 on the second day of the championships. The samples were always collected 10 min after the horse crossed the finish line, following the recommendations formulated by Bozovic et al. [32] and as in the study conducted by Munk et al. [5]. The samples were stored at +4 °C. Next, they were centrifuged for 10 min at 1500× *g*, and saliva was aspirated [33]. The levels of cortisol were assessed with the ELISA SLV2930 immunoassay (DRG Instruments GmbH, Marburg, Germany) for the determination of cortisol in saliva. The absorbance was measured using a BioTek^®^ EON microplate reader All samples were tested in duplicate.

### 2.3. Statistical Analyses

The normality of the data was verified with the Shapiro–Wilk test. Because one of the cortisol level variables did not have a normal distribution, nonparametric tests were used in the calculations. Salivary cortisol levels (baseline—T1, after arena assessment—T2, after endurance condition test—T3 and after Hucul path—T4) were characterized by giving mean values, standard deviation (SD), coefficient of variation (V), range and *p* value. The significance of differences between means was analyzed using Kruskal–Wallis ANOVA. The medians for the variables were compared in the graphs. Spearman’s R correlation coefficients were calculated for the association between cortisol levels and the results obtained by the mares in individual elements of the Hucul Horse Championships. Included were resting heart rate, the result of the arena assessment, race, i.e., endurance condition test, average speed of endurance, result of the Hucul path, average speed of Hucul path, the total result of the championship. The calculations were made in the Statistica 13.3 package.

## 3. Results

### 3.1. Cortisol Level Relative to the Age of Mares

The assessment of the cortisol level relative to age showed lower values in the younger (up to 9-year-old) mares. The level of cortisol determined in saliva ranged from 2.60 + 1.26 ng/mL (T1) to 10.40 + 8.27 ng/mL (T3) in mares up to 9 years of age, *p* value = 0.0001. In older mares, it was 2.86 ± 1.16 ng/mL and 10.51 ± 8.28 ng/mL, respectively, with *p* value = 0.0010 (Table 1). For both young and older mares, the coefficient of variation shows little to moderate dispersion, meaning that the mean characterizes the group well in terms of resting salivary cortisol levels, after arena evaluation, and after the Hucul path.

**Figure 2:**
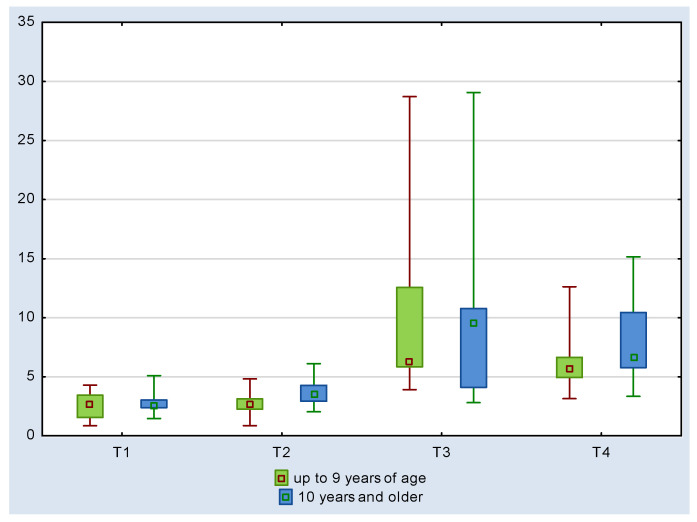
Cortisol level in mare saliva taking into account age, median, percentiles and Kruskal–Wallis test (≤9 years of age—KW-H(3.36) = 22.083; *p* = 0.0001; ≥10 years of age: KW-H(3.36) = 16.1772; *p* = 0.0010). The variables after the endurance–condition test are characterized by right-handed asymmetry. In contrast, half of the most typical data (box) are characterized by high similarity. Explanations as in Table 1.

However, within the age groups, there were significant differences between the levels of free cortisol in saliva determined after the consecutive competitions of the championships (Table 1 and Figure 2).

A positive correlation was found between resting heart rate (A) and cortisol levels after the breeding assessment (T2) in mares up to 9 years of age and in older mares. Furthermore, in the case of older mares, there is a negative correlation between resting heart rate (A) and the score obtained for the endurance condition test (C), and a positive one for the average speed in the endurance condition test (D). The correlation analysis in mares up to 9 years of age shows a negative correlation between the baseline cortisol level (T1) and the mean speed during the Hucul path (F) (−0.81, *p* = 0.04) and determined after breeding assessment (T2) (−0.71, *p* = 0.04). At the same time, in younger mares the result in the Hucul Horse Championships (G) was influenced by the score for the finished Hucul path (E). In older mares, the baseline cortisol level (T1) was negatively related to the score obtained for the finished Hucul path (E) (−0.67, *p* = 0.04), while positively related to the cortisol level after the Hucul path (T4). The score for the breeding assessment (B) was positively correlated with cortisol levels after this element of the championship (T2) (0.67, *p* = 0.04). The increase in cortisol level after the endurance–condition test (T3) was positively related to the level determined after the Hucul path (T4). (Table 2).

### 3.2. Cortisol Levels in the Saliva of Mares in Relation to the Number of Starts in the Qualification Paths

**Figure 3:**
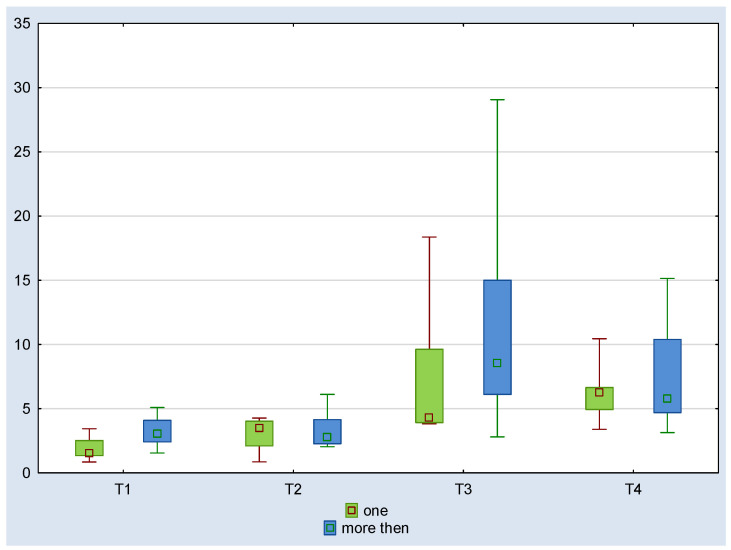
Free cortisol level in saliva of mares competing in one and more qualifying paths, median, percentiles and Kruskal–Wallis test (one: KW-H(3.24) = 13.94 *p* = 0.0030; more than one: KW-H(3.48) = 26.2968 *p* = 0.00001). The variables after the endurance–condition test are characterized by right-handed asymmetry. Explanations as in Table 1.

The analysis showed a significantly lower concentration of cortisol determined at baseline in mares starting only once in the qualification path (*p* = 0.0351). The level of cortisol determined after the arena assessment, Hucul path, and endurance–condition test was lower as well (Table 3). The increase in the level of cortisol determined after the subsequent elements of the championships was statistically significant in mares that competed in the qualification paths both only once and repeatedly (Table 3, Figure 3).

The analysis of the correlational relationships in the case of mares competing in a single qualifying path showed that the result of the championship (G) was influenced only by the score obtained for the completed Hucul path (E) (0.88, *p* = 0.02). However, the baseline cortisol level (T1) was positively correlated with the level determined after breeding assessment (T2). A high negative correlation was found between T1 and (F) (−0.94, *p* = 0.001). For mares competing in more than one qualifying path, the score obtained in the endurance–condition test (C) was positively correlated with the score in the championship (G) 0.80, *p* = 0.002) (Table 4).

## 4. Discussion

The evaluation of the performance value of the breeds included in the genetic resources protection programs is of great importance in the aspect of monitoring the quality of the population and thus of consolidating its specific and distinguishing features. The rules of the Hucul Horses Championships indicate, as a priority, the welfare of the horse during each element of the competition. Therefore, in a pilot study, an attempt was made to assess whether participation of horses in competitions causes stress, and that experience can reduce it. As reported by Ferlazzo et al. [34], exercise stress in sport horses is considered a physiological stressor, although it can also be considered a mental or psychogenic stressor. In competition horses, exercise stress depends on many factors. It results from individual predispositions, the level of preparation, and also the reaction to stress conditions. According to Ferlazzo et al. [35] in their study, they had difficulty differentiating between mental and physical effort. The authors concluded that changes in cortisol were associated with an increase in the intensity of competitive exercise. Horse participation in competition results in staying in unfamiliar stables or riding in close proximity to unfamiliar horses [34], which can be associated with the horse experiencing discomfort. The response to stressors is dependent on previous experience [36], but the individual response to stress is a personal function of the ability to perceive stress and control it [35]. The results of our own study do not confirm that participation in more Hucul horse competitions resulted in the acquisition of experience. Cortisol concentrations were higher in older mares and with a higher number of starts on qualifying trails. In our study, stress was assessed in horses that are actually difficult to consider “sporty” because their daily use is completely different. It was assumed that adaptation to the load may result from previous experience of exposure to an analogous load while competing in qualifying trails running in a similar formula.

According to the rules of the championships, the arrival of the competing teams takes place one day before the competition. However, we cannot exclude the influence of transport, a new environment, or the presence of other horses on the occurrence of a stress reaction in the mares studied. At the same time, this problem affects all horses participating in the competition, especially outside their home stable, which was confirmed by Peeters et al. [3]. In the present study, an increase in the cortisol level was detected after each phase of the championships. On the first day, it was almost four-fold higher after the endurance–condition test than the baseline cortisol level. Perhaps Hucul horses are not a breed predisposed to intensive load in a relatively short period of time, which they experience during the endurance condition test but also the Hucul path. This primitive mountain horse breed is extremely endurance [27,28,29]; however, due to the specific nature of mountainous areas, it moves mainly on a walk during equestrian mountain tourism. Perhaps in this case, the method of assessing the performance value of Hucul horses should be slightly verified, such that it really reflects the breed predispositions. Taking into account the presence of a circadian rhythm in horses [5,9,17], the present findings indicate a strong stress response in the mares assessed in the study. As reported by Fazio et al. [36], changes in cortisol levels can provide a good measure of qualitative and quantitative responses to stress and therefore provide an objective indicator of the effects of participation in the championship. As reported by Munk et al. [5], the circadian rhythm was maintained despite the increase in cortisol levels in a new environment and during competitions. The high level of cortisol in the saliva of the analyzed horses, compared to the baseline measured before the competitions, may therefore indicate that the designed test was an excessive burden. Similarly, on the second day of the competition (Hucul path in the morning), the level of free cortisol determined in horse saliva was over two-fold higher than the baseline level, and the differences were statistically significant (Table 1 and Table 3). This could be related to the relatively long recovery time after exercise. According to Golland et al. [37], analysis of selected hormones in four Standardbred geldings showed that maximal exercise disturbed plasma cortisol levels up to 24 h after exercise.

The correlation analysis did not give an unambiguous answer on the possible influence of cortisol level on the results in the case of mares up to 9 years old, because the final score was influenced by the result obtained on the Hucul path. However, in older mares, the cortisol level coefficient of rest was negatively related to the Hucul path completed score (Table 2). Similarly, no correlation was confirmed with the results obtained in the individual elements of the championship and ultimately with the final score obtained by the competing mares. Perhaps we should look at the mobilizing role of stress in horses that are regularly trained and compete differently from those that are subjected to high stress only during performance tests. Peeters et al. [3] pointed out the positive influence of stress on performance in the case of competitions held in home conditions. At the same time, he pointed out that a change of environment and a new environment may result in an increased stress response. Therefore, reducing aversive stimuli can be important for athletic performance and thus for the well-being of horses [34]. According to Bartolome and Cockram [38], stress can have positive but also negative effects on the body of a sport horse. It helps the animal cope with short-term stressors, which may improve performance in some circumstances but may worsen it in others. This suggestion is supported by the study by Negro et al. [39] investigating the effect of stress levels on sport performance during trotting races. The authors, when analyzing eye temperature, highlighted that precompetition stress affected the performance of horses in competition. Similarly, an increase in cortisol levels after intense physical activity associated with participation in competitions was reported by Becker-Birck et al. [40]. In Arabian pureblood horses, the highest increase was noted after endurance competitions [18], but it was lower in best-ranked animals.

Hucul horses are a primitive breed whose characteristics are determined by the mountainous environment of the Eastern Carpathians. They have historically been used as a reliable means of transport in difficult terrain. These horses are considered gentle and clever with high endurance, which contributes to their popularity [28,29]. As specified in the current regulations, in addition to the evaluation of the breeding value, the assessment of the performance value should include a start in the Hucul path and endurance–condition tests [24]. Many owners of Hucul horses enroll their animals for multiple assessments of the performance value in consecutive years [41] and for several qualifications each year to score the best ranking results and the right to start in the Championships for Hucul Horses. These “tests” are not de facto related to sport competitions offering specific benefits; they only confirm the performance value of this breed. They are attended by amateur competitors, often young and with insufficient awareness of the relationships between intense effort and welfare. Moreover, they often assume that horses gain experience with subsequent starts and achieve habituation through the repetition of potentially stressful factors, thus excluding their negative role in induction of stress. However, as reported by Grissom and Bhatnagar [42], the habituation of HPA activity in response to stressors depends on many additional factors. The awareness of the importance of a high level of welfare should be a priority, regardless of the breed and use of the horse or the rank and type of competitions [43,44,45,46]. As reported by Witkowska-Piłaszewicz et al. [18], participation in competitions is stressful to horses, which is indicated by higher cortisol levels than the post-training levels. Similarly, Becker-Birck et al. [40] demonstrated an increase in the cortisol release and heart rate during three-day competitions. The study involved horses that started often, which did not reduce their stress response. In a study conducted by Munk et al. [5], a higher concentration of cortisol was found in horses competing in dressage than in show jumping.

As reported by Sauer et al. [47], stress resistance may be related to the animal breed as well as maintenance and use of the animal. The high level of cortisol in the examined horses may be related to these indicators, which suggests a need for further research in this field. Conversely, the Hucul mares that took part in the Polish Championships for Hucul horses were mainly used for recreation, and the participation in the competitions may have been a source of severe stress. Taking into account the maintenance and use regime recommended for the breed for the preservation of its distinctive traits, further studies are planned. This will include, among other things, the maintenance system for competition horses, the type of work they do, their training methods, and workload. As this was a pilot study, the scope of further research is subject to further consideration. Taking into account that the evaluated horses showed elevated cortisol concentrations during starts, it would be worthwhile in further studies to also consider the evaluation of behavioral or physiological indicators, e.g., eye temperature, fecal cortisol metabolites, easy to measure and not requiring saliva or blood collection. The association between salivary cortisol concentrations and the aforementioned parameters was confirmed in studies by Janczarek et al. [6], Pawluski et al. [19], and Valera et al. [22]. Janczarek et al. [6], in their study on the connection between psychosomatic condition evaluation and salivary cortisol concentration in Purebred Arabian horses, found that this assessment can only be used based on an analysis of certain elements assessed immediately after exercise or during the first minutes of restitution. The authors cited the way of sweating and some organoleptic properties of sweat. The authors are aware of the limitations of the study conducted. They result from several factors beyond the authors’ control. The championships have a voluntary character, and horse owners usually approach the conducted research quite distrustfully. During the 2019 Hucul Horses Championships, they did not give their consent to implant devices that monitored the heart rate of horses during the competition, nor did they give their consent to have their blood drawn. Another limitation in obtaining data is the number of horses competing. The right to start in the Hucul Horse Championships is given to the 50 horses with the highest scores in the ranking updated after each qualifying path. However, not all horse owners decide to participate in the competition. The amount of the prizes is not encouraging, the participation and taking a high position of the horse is rather prestigious. Not all horses also successfully complete the various elements of the championship. This limits the possibility of obtaining a sufficiently large number of attempts.

While appreciating the importance of assessment of the breeding and performance value of horses, it is worth considering the possible association of high levels of stress with multiple starts in competitions, which may exert a negative impact on the welfare of these animals.

## 5. Conclusions

The present study has shown that the participation of horses in the competitions was not accompanied by a lower stress level, despite the previous experience. In older mares (over 10 years) who underwent the qualification path for several years and more than once in the study year, the level of free cortisol in saliva was clearly higher. Their stress reaction developed in response to the first element of the Polish Championships for Hucul Horse, i.e., the breeding assessment. Similarly, the reaction to the consecutive competitions was clearly worse in this group. The effect of cortisol levels on the final performance of horses in competition has not been confirmed. These preliminary research results may indicate the need for critical assessment of the application of multiple performance tests in horses that are often improperly prepared for intense effort.

## Figures and Tables

**Table 1:** Cortisol level relative to mares’ age (ng/mL).

Mares		T1	T2	T3	T4	*p* Value
Total	n	18	18	18	18	
	mean ± SD	2.73 ± 1.18	3.19 ± 1.28	10.46 ± 8.03	7.10 ± 3.41	-
	range	0.85–5.10	0.86–6.12	2.81–29.07	3.14–15.15	-
≤9 years	n	9	9	9	9	
	mean ± SD	2.60 ± 1.26 ^AD^	2.74 ± 1.15 ^D^	10.40 ± 8.27 ^C^	6.49 ± 3.03 ^BC^	0.0001
	V %	48.5	42.0	79.5	46.7	
	range	0.85–4.30	0.86–4.80	3.91–28.72	3.14–12.62	
≥10 years	n	9	9	9	9	
	mean ± SD	2.86 ± 1.16 ^A^	3.64 ± 1.31 ^ABC^	10.51 ± 8.28 ^C^	7.71 ± 3.84 ^B^	0.0010
	V %	40.5	35.9	78.8	49.8	
	range	1.47–5.10	2.03–6.12	2.87–29.07	3.35–15.15	
	*p* Value	0.6571	0.1420	0.9790	0.4671	

Saliva samples were collected: T1—baseline samples prior to the competition; T2—after breeding assessment; T3—after the endurance–condition test; T4—after the Hucul path. Means in rows marked with different letters statistically differ.

**Table 2:** Spearman R correlation coefficient and *p* value; above—up to 9-year-old mares; below—10-year-old and older mares.

	A	T1	B	T2	C	D	T3	E	F	T4	G
A		ns	ns	0.82 *p* = 0.01	ns	ns	ns	ns	ns	ns	ns
T1	ns		ns	ns	ns	ns	ns	ns	−0.81 *p* = 0.01	ns	ns
B	ns	ns		ns	ns	ns	ns	ns	ns	ns	ns
T2	0.67 *p* = 0.04	ns	0.67 *p* = 0.04		ns	ns	ns	ns	−0.71 *p* = 0.04	ns	ns
C	−0.83 *p* = 0.01	ns	ns	0.69 *p* = 0.04		ns	ns	ns	ns	ns	ns
D	0.76 *p* = 0.02	ns	ns	ns	ns		ns	ns	ns	ns	ns
T3	ns	ns	ns	ns	ns	ns		ns	ns	ns	ns
E	ns	−0.67 *p* = 0.04	ns	ns	ns	ns	ns		ns	ns	0.88 *p* = 0.001
F	ns	ns	ns	ns	ns	ns	ns	ns		ns	ns
T4	ns	0.67 *p* = 0.04	ns	ns	ns	ns	0.67 *p* = 0.04	ns	ns		ns
G	ns	ns	ns	ns	ns	ns	ns	ns	ns	ns	

A—resting heart rate, T1—cortisol baseline, B-breeding assessment—points, T2—cortisol after breeding assessment, C—endurance condition test—points, D—average speed of endurance condition test, T3—cortisol after endurance condition test, E—Hucul path—points, F—average speed of Hucul path, T4—cortisol after Hucul path, G—the result of the championship—points; ns—no significant relationship.

**Table 3:** Cortisol levels in Hucul mare saliva (ng/mL) relative to their number of starts in the qualification paths.

Number of Starts		T1	T2	T3	T4	*p* Value
≥2 starts	n	12	12	12	12	
	mean ± SD	3.16 ± 1.07 ^AD^	3.26 ± 1.30 ^D^	11.99 ± 8.74 ^C^	7.49 ± 3.87 ^B^	0.0000
	V %	33.9	39.9	72.9	51.7	
	range	1.55–5.10	2.03–6.12	2.81–29.07	3.14–15.15	
=1 starts	n	6	6	6	6	
	mean ± SD	1.87 ± 0.94 ^A^	3.04 ± 1.35 ^ABC^	7.38 ± 5.82 ^C^	6.33 ± 2.36 ^B^	0.0030
	V %	50.3	44.4	78.7	37.3	
	range	0.85–3.44	0.86–4.27	3.81–18.36	3.39–10.34	
	*p* Value	0.0351	0.7715	0.3297	0.5703	

Saliva samples were collected: T1—baseline samples prior to the competition; T2—after breeding assessment; T3—after the endurance–condition test; T4—after the Hucul path. Means in rows marked with different letters statistically differ.

**Table 4:** Spearman R correlation coefficient and *p* value for number of qualifications; above—1 start; below—2 and more starts.

	A	T1	B	T2	C	D	T3	E	F	T4	G
A		ns	ns	0.88 *p* = 0.02	ns	ns	ns	ns	ns	ns	ns
T1	ns		ns	0.83 *p* = 0.04	ns	ns	ns	ns	−0.94 *p* = 0.001	ns	ns
B	ns	ns		ns	ns	ns	ns	0.83 *p* = 0.04	ns	ns	ns
T2	ns	ns	ns		ns	ns	ns	ns	ns	ns	ns
C	ns	ns	ns			−0.96 *p* = 0.001	ns	ns	ns	ns	ns
D	ns	ns	ns	ns	ns		ns	ns	ns	ns	ns
T3	ns	ns	ns	ns	ns	ns		ns	ns	ns	ns
E	ns	ns	ns	ns	ns	ns	ns		ns	ns	0.88 *p* = 0.02
F	ns	ns	ns	ns	ns	ns	ns	ns		ns	ns
T4	ns	ns	ns	ns	ns	ns	0.83 *p* = 0.001	ns	ns		ns
G	ns	ns	ns	ns	0.80 *p* = 0.002	ns	ns	ns	ns	ns	

A—resting heart rate, T1—cortisol baseline, B-breeding assessment—points, T2—cortisol after breeding assessment, C—endurance–condition test—points, D—average speed of endurance–condition test, T3—cortisol after endurance–condition test, E—Hucul path—points, F—average speed of Hucul path, T4—cortisol after Hucul path, G—the result of the championship—points; ns—no significant relationship.

## Data Availability

The following are available online at: https://https://repozytorium.ur.edu.pl/handle/item/6905; Arena assessment, endurance–condition test and Hucul path (accessed on 15 November 2021). The details data are available on reasonable request from the corresponding author. The Hucul Breeding Programme is available on the website of the Polish Horse Breeders’ Association https://www.pzhk.pl/wp-content/uploads/pr-hodow-hc-2020-08-10.pdf (accessed on 19 November 2021).
